# Stabilization period before capturing an ultra-short vagal index can be shortened to 60 s in endurance athletes and to 90 s in university students

**DOI:** 10.1371/journal.pone.0205115

**Published:** 2018-10-08

**Authors:** Jakub Krejčí, Michal Botek, Andrew J. McKune

**Affiliations:** 1 Department of Natural Sciences in Kinanthropology, Faculty of Physical Culture, Palacký University Olomouc, Olomouc, Czech Republic; 2 Faculty of Health, Discipline of Sport and Exercise Science, UC-Research Institute for Sport and Exercise, University of Canberra, Canberra, Australia; 3 School of Health Sciences, Discipline of Biokinetics, Exercise and Leisure Sciences, University of KwaZulu-Natal, Durban, South Africa; University of Bourgogne France Comté, FRANCE

## Abstract

**Purpose:**

To find the shortest, acceptable stabilization period before recording resting, supine ultra-short-term Ln RMSSD and heart rate (HR).

**Method:**

Thirty endurance-trained male athletes (age 24.1 ± 2.3 years, maximal oxygen consumption (VO_2_max) 64.1 ± 6.6 ml·kg^-1^·min^-1^) and 30 male students (age 23.3 ± 1.8 years, VO_2_max 52.8 ± 5.1 ml·kg^-1^·min^-1^) were recruited. Upon awaking at home, resting, supine RR intervals were measured continuously for 10 min using a Polar V800 HR monitor. Ultra-short-term Ln RMSSD and HR values were calculated from 1-min RR interval segments after stabilization periods from 0 to 4 min in 0.5 min increments and were compared with reference values calculated from 5-min segment after 5-min stabilization. Systematic bias and intraclass correlation coefficients (ICC) including 90% confidence intervals (CI) were calculated and magnitude based inference was conducted.

**Results:**

The stabilization periods of up to 30 s for athletes and up to 60 s for students showed positive (possibly to most likely) biases for ultra-short-term Ln RMSSD compared with reference values. Stabilization periods of 60 s for athletes and 90 s for students showed trivial biases and ICCs were 0.84; 90% CI 0.72 to 0.91, and 0.88; 0.79 to 0.94, respectively. For HR, biases were trivial and ICCs were 0.93; 0.88 to 0.96, and 0.93; 0.88 to 0.96, respectively.

**Conclusion:**

The shortest stabilization period required to stabilize Ln RMSSD and HR was set at 60 s for endurance-trained athletes and 90 s for university students.

## Introduction

Analysis of heart rate variability (HRV) has provided a non-invasive method for evaluating cardiac autonomic regulation [1,2]. In sports science, important applications of HRV analysis include monitoring responses to training loads [3–7], detection of overreaching signs [8,9], and HRV-guided training [10–13]. It is necessary for HRV analysis to record RR intervals for a sufficient period. A 5-min recording period was recommended as the standard for short-term HRV analysis [2]. In addition, a resting recording should be started when RR intervals have stabilized. Therefore, a stabilization period is required before the start of the recording. However, guidelines [2] did not provide recommendations for choosing the stabilization period. Various stabilization periods have been used in the literature: 0 min [10,11], 1 min [6,9], 2 min [7], 3 min [3], 4 min [8], and 5 min [4]. Alternatively, the stabilization period was automatically selected based on stable heart rate (HR) detection [5]. It is clear that the stabilization period is not sufficiently standardized in sports science literature.

Coaches who implement HRV analysis as part of a training strategy may struggle with low athlete compliance relating to regular measurement, which should be performed on a daily basis [14,15]. Daily HRV analysis using a 5-min stabilization period and 5-min recording period takes 70 min per week, which may be considered time-consuming, with athletes gradually becoming less compliant and not providing regular HRV data. We have often been asked by athletes to make HRV analysis more time-effective. This challenge can be solved in several ways: a) reducing the number of HRV measures per week, b) shortening the recording period, and c) shortening the stabilization period. Previously, Plews et al. [16] showed that three HRV analyses per week were sufficient for trained athletes but five analyses per week were necessary for recreational athletes. Another study [6] found that three analyses per week were sufficient in the supine position, but discrepancies were found in the standing position. Therefore, five HRV analyses per week were recommended for standing HRV [6].

The root mean square of successive differences between adjacent RR intervals (RMSSD) has been regarded as an index of vagal activity [1,14] and considered to be a more reliable marker of an athlete’s training status compared with high-frequency power (HF) [17]. Some studies [12,13] have used RMSSD directly, others [3–6,8,9] used a derived variable, Ln RMSSD, which is calculated from RMSSD using natural logarithm. The reason for the logarithmic transformation is the correction of the skewed probability distribution of RMSSD [9,18]. A primary advantage of Ln RMSSD, compared with HF, is that it can be calculated from 10 s RR recordings [19]. However, Esco & Flatt [19] recommended a 1-min recording period as a compromise between sufficient reliability and time demand. Recently, it was reported that ultra-short-term (1 min) time domain indexes may be useful surrogates of the short-term (5 min) frequency domain indexes in athletes [20]. In relation to training practice, ultra-short-term Ln RMSSD showed a sufficient sensitivity to changes induced by training [21].

Plews et al. [22] showed that Ln RMSSD assessment alone may be misleading due to possible presence of the parasympathetic saturation phenomenon, specifically in athletes with very high cardiac vagal activity. The saturation phenomenon causes the relationship between the vagal index and the average RR to be quadratic rather than linear and is likely caused by the nonlinear dose response of the sinoatrial node to the acetylcholine secreted by vagal nerve ending [23]. The cut point between the linear and the saturation area was individual and ranged around 50 beats/min [23]. When an athlete experiences this saturation phenomenon, it is not possible to track changes in the vagal activity simply based on changes in the vagal index [22]. For example, an athlete during different stages of training may have a high cardiac vagal activity manifested by low resting supine HR (e.g. 40 beats/min) on one day but the Ln RMSSD value may be similar or even lower compared to another day in which the athlete may have a lower vagal activity and higher HR (e.g. 60 beats/min). Therefore, it is recommended that the evaluation of vagal activity is performed together with HR assessment in athletes.

It was shown that the stabilization period can be shortened from 5 min to 1 min [24,25]. However, based on our opinion these studies had the following limitations: a) During the stabilization period, the ECG electrodes were placed on the participant, which may have disturbed RR intervals and affected the HRV. b) No analysis of HR stabilization was provided, and therefore the saturation phenomenon could not be evaluated.

Therefore, the aim of this study was to find the shortest, acceptable stabilization period before capturing ultra-short-term Ln RMSSD and HR in the supine position.

## Methods

### Participants

The study protocol was approved by the Ethics Committee of the Faculty of Physical Culture, Palacký University Olomouc (reference number 14/2015) and was done in accordance with the Declaration of Helsinki. After comprehensive explanation of the study, all participants provided written informed consent. Inclusion criteria were as follows: male, aged 20 to 29 years, non-smoker, and taking no medication. Prior to participation in the study, participants underwent resting 10-lead electrocardiogram (ECG) examination and blood pressure measurement. The exclusion criteria included a pathological ECG pattern and hypertension (> 140/90 mmHg). This study included two groups. The first group consisted of 30 Czech national level endurance male athletes (10 skyrunners, 8 road cyclists, and 12 cross-county skiers) and the second group consisted of 30 male university students. Characteristics of participants are presented in Table 1.

**Table 1:** Statistics of the studied groups.

Variable	Athletes	Students	Chances +/tri/-	Inference
	Mean ± SD	Mean ± SD	(%)	
Age (years)	24.1 ± 2.3	23.3 ± 1.8	74/24/2	possibly positive
Body mass (kg)	74.5 ± 6.6	79.1 ± 5.3	0/1/99	very likely negative
Body height (cm)	179.9 ± 3.8	183.7 ± 4.1	0/0/100	most likely negative
VO_2_max (ml·kg^-1^·min^-1^)	64.1 ± 6.6	52.8 ± 5.1	100/0/0	most likely positive
Ln RMSSD (ms)	4.43 ± 0.50	4.30 ± 0.45	60/36/4	possibly positive
RMSSD (ms)	95 ± 54	81 ± 35	67/30/3	possibly positive
HR (beats·min^-1^)	50.8 ± 6.2	56.3 ± 6.4	0/1/99	very likely negative

SD = standard deviation; Chances = chances that the true value of difference (x_athletes_−x_students_) is substantially positive, trivial, or substantially negative; VO_2_max = maximal oxygen consumption; Ln RMSSD = natural logarithm of root mean square of successive differences between adjacent RR intervals; RMSSD = root mean square of successive differences between adjacent RR intervals; HR = heart rate.

### Procedures

Body mass, body height and maximal oxygen consumption (VO_2_max) were determined in a laboratory a week before HRV recording and were used for descriptive purposes only. Body mass and height were measured using the Soehnle 7307 scale (Leifheit, Nassau, Germany). VO_2_max was determined during an incremental running test on the treadmill (Valiant Plus, Lode, Groningen, Netherlands). The protocol consisted of a 4 min warm-up (2 min at 8 km.h^-1^ with 0% elevation and then 2 min at the same speed with 5% elevation) followed by an increase in speed to 10 km.h^-1^ with 5% elevation for 1 min. From this point, at each minute, the speed was increased by 1 km.h^-1^, keeping elevation the same, up to 16 km.h^-1^. Then the speed was maintained and only the elevation increased by 2.5% per minute until exhaustion. Ventilation and gas exchange were recorded breath by breath and averaged to 30 s by Blue Cherry system (Geratherm Respiratory, Bad Kissinger, Germany). The criteria for attaining VO_2_max was defined as reaching one of the following criteria: a) respiratory exchange ratio of >1.11, b) VO_2_ plateau defined as no increase in VO_2_ in response to an increase in work rate. VO_2_max was considered the highest VO_2_ value in the final 30 s of the test.

### HRV measurement

Participants were asked to refrain from the consumption of caffeine or alcohol and to avoid strenuous exercise for 24 h prior to HRV measurement. Each participant measured his RR intervals at home using a Polar V800 HR monitor (Polar, Kempele, Finland) which was showed as valid RR interval measurement tool [26]. Participants were instructed to leave the HR monitor by their bedside in the evening. In the morning after awakening and emptying their bladder, they were instructed to put on the monitor and ECG chest strap, prepare the monitor for recording and adopt a supine position on the bed. Immediately upon lying down, they started RR recording. The participants remained supine for at least 10 min, after which they were asked to stop and save their RR recordings. In the present study, participants were allowed to breathe spontaneously during the RR recording. We did not use paced breathing as a standardization procedure because it was shown that voluntary control of breathing reduced spectral power in the respiratory frequency region [27]. In this context, it was showed that RMSSD was more resistant to changes in breathing rate compared with HF [28].

RR recordings were transferred to a computer using the Polar Flow cloud service and were further analyzed using a custom program written in MATLAB language (MathWorks, Natick, MA). Artifacts (ectopic beats, missing beats, etc.) were identified by visual inspection of RR intervals and simply deleted because the deletion method provided the best overall performance [29].

Ultra-short-term values of Ln RMSSD, RMSSD, and average values of HR were calculated from 1-min segments using various stabilization periods. Stabilization periods varied from 0 to 4 min with step interval increase of 0.5 min which yielded 9 periods in total. The first segment used no stabilization period (0 min) and included RR intervals between 0 and 1 min after the start of RR recording. The second segment used a stabilization period of 0.5 min and included RR intervals between 0.5 and 1.5 min. This procedure continued stepwise and finally the 9th segment used a stabilization period of 4 min and included RR intervals between 4 and 5 min. Reference values of Ln RMSSD, RMSSD, and HR were calculated from the second half of the 10-min RR recording, resulting in a 5-min segment of RR intervals from 5 to 10 min.

### Statistical analysis

Data are presented as arithmetic mean ± standard deviation (SD). Anthropological and physiological variables (Table 1) of endurance athletes were compared with the values of university students using a two-sample t-test. As an index of vagal activity we prefer to use Ln RMSSD rather than RMSSD. However, literature is not consistent whether or not a logarithmic transformation should be used. Therefore, we performed a statistical analysis for both Ln RMSSD and RMSSD. Systematic bias of Ln RMSSD, RMSSD, and HR measurement was calculated as the ultra-short-term value minus reference value. Systematic bias was compared to zero using an one-sample t-test. Typical error (TE) was calculated using the formula $\mathrm{TE}={\mathrm{SD}}_{\mathrm{diff}}/\sqrt{2}$, where SD_diff_ is standard deviation calculated from differences between ultra-short-term and reference values [30]. Agreement between the ultra-short-term and reference values was evaluated using an intraclass correlation coefficient (ICC). The appropriate formula for calculating ICC was chosen using a decision tree [31] and the formula labeled as ICC(A, 1) was chosen.

Because null-hypothesis significance testing (NHST) is not able to distinguish between a trivial effect and an insufficient sample size, we used magnitude-based inference (MBI) [32]. MBI was based on calculating a 90% confidence interval (CI) that defines a range representing the uncertainty in the true value. A three-level scale: substantially positive, trivial, and substantially negative, was defined by the smallest worthwhile change (SWC). Chances that the true value was substantially positive, trivial, and substantially negative, were calculated by comparing the CI to the three-level scale. If the chance of substantially positive or substantially negative was simultaneously >5%, the true value was deemed as unclear. Otherwise the chances were labeled quantitatively as follows: 25–75%, possibly; 75–95%, likely; 95–99.5%, very likely; and >99.5%, most likely [33]. We used a spreadsheet [34] to convert a p-value into CI and chances. The SWC for comparing anthropological and physiological variables was set to 0.2 of the pooled standard deviation calculated from between-individual standard deviations of both groups. The SWC for systematic bias was set to 0.2 of the standard deviation calculated from reference values of Ln RMSSD, RMSSD, or HR. Only very large (.70–.89) [33] and extremely large (≥.90) [33] ICCs were considered meaningful, so the SWC for ICC was set to 0.70. Requirements for accepting a stabilization period as sufficient were as follows: a) the systematic bias is trivial; b) lower limit of 90% CI for ICC is >.70, within MBI concept this requirement is equivalent to ICC is at least very likely positive. Conventional statistical analysis using NHST and 95% CI is presented in the S1–S4 Tables.

## Results

Athletes, compared with the students, demonstrated most likely higher VO_2_max, possibly higher both Ln RMSSD and RMSSD, and very likely lower HR (Table 1). For athletes, the shortest stabilization period that met our requirements described in the method section was 1.0 min for both Ln RMSSD (Table 2) and RMSSD (Table 3) and 0.5 min for HR (Table 4). Therefore, for the simultaneous measurement of Ln RMSSD and HR, the shortest stabilization period was 1.0 min. Stabilization periods from 1.5 to 4.0 min also met the requirements. For students, the shortest stabilization period was 1.5 min for both Ln RMSSD (Table 2) and RMSSD (Table 3) and 1.0 min for HR (Table 4). For the simultaneous measurement of Ln RMSSD and HR, the shortest stabilization period was 1.5 min. Stabilization periods ranging from 2.0 to 4.0 min also met the requirements.

**Table 2:** Comparison of the Ln RMSSD values that were calculated from a 1-min segment after various stabilization periods (SP) with reference Ln RMSSD values that were calculated from 5-min segments after a 5-min stabilization period.

SP	Mean ± SD	Bias; ±90% CL	Chances +/tri/-	Inference	TE	ICC (90% CI)	Chances +/tri/-	Inference
(min)	(ms)	(ms)	(%)		(ms)		(%)	
Athletes (n = 30)								
0.0	4.58 ± 0.52	0.15; ±0.09	82/18/0	likely positive	0.21	0.79 (0.61 to 0.89)	87/13/0	likely positive
0.5	4.55 ± 0.50	0.12; ±0.10	65/35/0	possibly positive	0.22	0.78 (0.62 to 0.88)	84/16/0	likely positive
1.0	4.45 ± 0.59	0.02; ±0.10	9/89/2	likely trivial	0.22	0.84 (0.72 to 0.91)	97/3/0	very likely positive
1.5	4.42 ± 0.60	-0.01; ±0.08	1/97/2	very likely trivial	0.17	0.90 (0.83 to 0.95)	100/0/0	most likely positive
2.0	4.42 ± 0.62	-0.01; ±0.09	3/92/5	likely trivial	0.21	0.86 (0.76 to 0.92)	99/1/0	very likely positive
2.5	4.46 ± 0.62	0.03; ±0.08	9/90/1	likely trivial	0.19	0.89 (0.80 to 0.94)	100/0/0	most likely positive
3.0	4.43 ± 0.58	0.00; ±0.07	1/98/1	very likely trivial	0.16	0.92 (0.85 to 0.96)	100/0/0	most likely positive
3.5	4.37 ± 0.57	-0.06; ±0.08	0/82/18	likely trivial	0.19	0.88 (0.78 to 0.93)	99/1/0	very likely positive
4.0	4.36 ± 0.60	-0.07; ±0.07	0/75/25	possibly trivial	0.17	0.90 (0.83 to 0.95)	100/0/0	most likely positive
Ref	4.43 ± 0.50							
Students (n = 30)								
0.0	4.59 ± 0.46	0.29; ±0.10	100/0/0	most likely positive	0.23	0.62 (0.19 to 0.81)	22/78/0	likely trivial
0.5	4.54 ± 0.50	0.24; ±0.10	99/1/0	very likely positive	0.22	0.71 (0.35 to 0.86)	53/47/0	possibly positive
1.0	4.45 ± 0.51	0.15; ±0.08	88/12/0	likely positive	0.18	0.82 (0.63 to 0.91)	93/7/0	likely positive
1.5	4.36 ± 0.47	0.06; ±0.07	26/74/0	possibly trivial	0.15	0.88 (0.79 to 0.94)	100/0/0	most likely positive
2.0	4.33 ± 0.48	0.03; ±0.07	7/93/0	likely trivial	0.16	0.89 (0.80 to 0.94)	100/0/0	most likely positive
2.5	4.33 ± 0.52	0.03; ±0.08	9/90/1	likely trivial	0.18	0.87 (0.78 to 0.93)	99/1/0	very likely positive
3.0	4.30 ± 0.53	0.00; ±0.07	2/96/2	very likely trivial	0.16	0.90 (0.82 to 0.94)	100/0/0	most likely positive
3.5	4.29 ± 0.53	-0.01; ±0.07	1/96/3	very likely trivial	0.15	0.91 (0.83 to 0.95)	100/0/0	most likely positive
4.0	4.26 ± 0.52	-0.04; ±0.07	0/90/10	likely trivial	0.15	0.90 (0.82 to 0.95)	100/0/0	most likely positive
Ref	4.30 ± 0.45							

SD = standard deviation; Bias = mean difference between the 1-min segment value and reference value; CL = confidence limit; Chances = chances that the true value of bias or ICC is substantially positive, trivial, or substantially negative; TE = typical error; ICC = intraclass correlation coefficient; CI = confidence interval.

**Table 3:** Comparison of the RMSSD values that were calculated from a 1-min segment after various stabilization periods (SP) with reference RMSSD values that were calculated from 5-min segments after a 5-min stabilization period.

SP	Mean ± SD	Bias; ±90% CL	Chances +/tri/-	Inference	TE	ICC (90% CI)	Chances +/tri/-	Inference
(min)	(ms)	(ms)	(%)		(ms)		(%)	
Athletes (n = 30)								
0.0	112 ± 63	16; ±10	81/19/0	likely positive	23	0.81 (0.65 to 0.90)	92/8/0	likely positive
0.5	107 ± 58	12; ±10	57/43/0	possibly positive	24	0.81 (0.67 to 0.89)	91/9/0	likely positive
1.0	102 ± 66	7; ±9	21/79/0	likely trivial	20	0.89 (0.80 to 0.94)	100/0/0	most likely positive
1.5	100 ± 67	4; ±7	6/94/0	likely trivial	15	0.94 (0.88 to 0.96)	100/0/0	most likely positive
2.0	101 ± 70	5; ±9	15/85/0	likely trivial	20	0.90 (0.82 to 0.94)	100/0/0	most likely positive
2.5	105 ± 71	9; ±9	38/62/0	possibly trivial	20	0.89 (0.81 to 0.94)	100/0/0	most likely positive
3.0	99 ± 61	3; ±7	3/97/0	very likely trivial	15	0.93 (0.88 to 0.96)	100/0/0	most likely positive
3.5	93 ± 58	-2; ±8	0/95/5	likely trivial	19	0.89 (0.81 to 0.94)	100/0/0	most likely positive
4.0	93 ± 61	-2; ±6	0/99/1	very likely trivial	13	0.95 (0.91 to 0.97)	100/0/0	most likely positive
Ref	95 ± 54							
Students (n = 30)								
0.0	108 ± 45	27; ±11	100/0/0	most likely positive	24	0.52 (0.15 to 0.74)	6/94/0	likely trivial
0.5	105 ± 47	24; ±9	100/0/0	most likely positive	21	0.64 (0.27 to 0.82)	30/70/0	possibly trivial
1.0	96 ± 48	15; ±8	95/5/0	very likely positive	19	0.74 (0.51 to 0.86)	68/32/0	possibly positive
1.5	87 ± 37	6; ±6	34/66/0	possibly trivial	13	0.87 (0.77 to 0.93)	99/1/0	very likely positive
2.0	84 ± 38	3; ±5	12/88/0	likely trivial	12	0.89 (0.80 to 0.94)	100/0/0	most likely positive
2.5	86 ± 43	5; ±7	31/69/0	possibly trivial	16	0.84 (0.72 to 0.91)	96/4/0	very likely positive
3.0	84 ± 42	3; ±6	12/87/1	likely trivial	14	0.86 (0.76 to 0.92)	99/1/0	very likely positive
3.5	83 ± 41	2; ±6	7/92/1	likely trivial	13	0.89 (0.80 to 0.94)	100/0/0	most likely positive
4.0	81 ± 42	0; ±6	2/95/3	likely trivial	14	0.88 (0.78 to 0.93)	99/1/0	very likely positive
Ref	81 ± 35							

SD = standard deviation; Bias = mean difference between the 1-min segment value and reference value; CL = confidence limit; Chances = chances that the true value of bias or ICC is substantially positive, trivial, or substantially negative; TE = typical error; ICC = intraclass correlation coefficient; CI = confidence interval.

**Table 4:** Comparison of heart rate (HR) values that were calculated from a 1-min segment after various stabilization periods (SP) with reference HR values that were calculated from 5-min segments after a 5-min stabilization period.

SP	Mean ± SD	Bias; ±90% CL	Chances +/tri/-	Inference	TE	ICC (90% CI)	Chances +/tri/-	Inference
(min)	(ms)	(ms)	(%)		(ms)		(%)	
Athletes (n = 30)								
0.0	53.3 ± 7.5	2.6; ±1.2	97/3/0	very likely positive	2.7	0.79 (0.54 to 0.90)	86/14/0	likely positive
0.5	50.6 ± 7.1	-0.2; ±1.0	1/95/4	likely trivial	2.2	0.89 (0.81 to 0.94)	100/0/0	most likely positive
1.0	50.2 ± 6.9	-0.6; ±0.7	0/94/6	likely trivial	1.7	0.93 (0.88 to 0.96)	100/0/0	most likely positive
1.5	50.0 ± 6.9	-0.8; ±0.7	0/88/12	likely trivial	1.5	0.94 (0.89 to 0.97)	100/0/0	most likely positive
2.0	50.0 ± 7.0	-0.8; ±0.7	0/83/17	likely trivial	1.6	0.93 (0.88 to 0.97)	100/0/0	most likely positive
2.5	50.1 ± 6.6	-0.6; ±0.6	0/95/5	very likely trivial	1.3	0.95 (0.91 to 0.97)	100/0/0	most likely positive
3.0	50.3 ± 6.3	-0.5; ±0.7	0/96/4	very likely trivial	1.6	0.93 (0.87 to 0.96)	100/0/0	most likely positive
3.5	50.7 ± 6.7	-0.1; ±0.8	0/99/1	very likely trivial	1.8	0.93 (0.87 to 0.96)	100/0/0	most likely positive
4.0	50.9 ± 7.1	0.1; ±0.9	2/97/1	very likely trivial	2.0	0.91 (0.84 to 0.95)	100/0/0	most likely positive
Ref	50.8 ± 6.2							
Students (n = 30)								
0.0	58.5 ± 6.3	2.2; ±1.7	82/18/0	likely positive	3.9	0.60 (0.36 to 0.76)	17/83/0	likely trivial
0.5	56.5 ± 6.5	0.2; ±1.5	12/82/6	unclear	3.4	0.72 (0.54 to 0.84)	59/41/0	possibly positive
1.0	55.2 ± 6.1	-1.1; ±0.9	0/65/35	possibly trivial	2.1	0.87 (0.77 to 0.93)	99/1/0	very likely positive
1.5	55.6 ± 6.1	-0.7; ±0.7	0/92/8	likely trivial	1.6	0.93 (0.88 to 0.96)	100/0/0	most likely positive
2.0	56.0 ± 5.9	-0.3; ±0.7	0/99/1	very likely trivial	1.5	0.94 (0.89 to 0.97)	100/0/0	most likely positive
2.5	56.0 ± 6.1	-0.2; ±0.6	0/100/0	most likely trivial	1.5	0.95 (0.90 to 0.97)	100/0/0	most likely positive
3.0	55.8 ± 6.2	-0.5; ±0.5	0/99/1	very likely trivial	1.2	0.96 (0.93 to 0.98)	100/0/0	most likely positive
3.5	55.7 ± 6.0	-0.5; ±0.5	0/99/1	very likely trivial	1.1	0.96 (0.93 to 0.98)	100/0/0	most likely positive
4.0	55.8 ± 6.2	-0.5; ±0.6	0/99/1	very likely trivial	1.3	0.96 (0.92 to 0.98)	100/0/0	most likely positive
Ref	56.3 ± 6.4							

SD = standard deviation; Bias = mean difference between the 1-min segment value and reference value; CL = confidence limit; Chances = chances that the true value of bias or ICC is substantially positive, trivial, or substantially negative; TE = typical error; ICC = intraclass correlation coefficient; CI = confidence interval.

## Discussion

The primary finding of this study was that a shortened RR interval measurement, consisting of a 1-min stabilization period and 1-min recording period, is an acceptable substitution for the traditional procedure that uses a 5-min stabilization period and 5-min recording period in endurance athletes. The shortened measurement protocol saves up to 80% of the time and it is proposed that the reduced time commitment will improve the attractiveness of HRV analysis for athletes, who require guided training load based on HRV analysis on daily basis [14,15] or at least three times a week [6,16].

To date, two studies [24,25] have also reported on the use of a shortened stabilization period. Both studies recommended a 1-min stabilization period prior recording ultra-short-term Ln RMSSD. This is in line with our finding but there are two methodological differences between these studies and the present study.

Firstly, both studies [24,25] did not define the stabilization period sufficiently. There were trivial effect sizes of 0.07 [24] and 0.03 [25] between the Ln RMSSD calculated from the first minute of the 10 min recording and Ln RMSSD calculated from the 5 min criterion segment. In addition, ICC was extremely large (.92) [24] or very large (.89) [25]. Based on the above outcomes, no stabilization period seemed to be necessary. However, before the start of RR interval recording, there was 1-min pause that served for ECG electrodes placement on the participant’s chest in the supine position [24] or for the checking of the ECG chest strap in the seated position [25]. It is debatable whether this 1 min pause could be considered as part of the stabilization period because the fastening of the ECG electrodes may disturb the participant relaxation, and consequently, may affect the HRV. Therefore, in the present study, the participants were required to place the Polar HR watch on the hand and fasten the ECG chest strap whilst standing. Following this, participants were instructed to lie down and to start with the RR recording. Based on this protocol, the beginning of the stabilization period corresponded with the end of the clinostatic maneuver. Our results showed that no stabilization period as well as a stabilization period of 30 s were insufficient, because the ultra-short-term Ln RMSSD values were likely and possibly, respectively, biased compared to the reference Ln RMSSD.

Secondly, Flatt & Esco [24] performed the RR recordings in a laboratory with controlled settings (quiet, dimly lit, controlled temperature and humidity) which are prerequisites for obtaining a valid HRV analysis [35]. However, regular HRV analysis in athletes is often performed at home for logistical reasons and we followed the RR recording procedure used in other recent studies [5,6,8,13]. Based on the current authors’ experience, RR recordings performed at home may not be as well standardized as those performed in laboratory environment. However, home HRV analysis is not influenced by a white-coat effect [36] and obtains ecologically valid information on cardiac autonomic regulation. For the reasons mentioned above, it is proposed that the present results are better applicable for HRV analysis performed at home. Pereira et al. [25] performed the RR recordings in a gym, where the environmental conditions were not specified.

In this study, ICCs for Ln RMSSD after stabilization periods from 1 min to 4 min ranged from 0.84 to 0.92. Pereira et al. [25] reported ICCs in the range of 0.86 to 0.94 and we consider this range to be similar to the present findings. Flatt & Esco [24] reported ICCs in the range of 0.92 to 0.97 and this range is somewhat higher than the present study. These results can be explained by the fact that a lab environment can be standardized more consistently than at home or in a gym. Our study involved 30 male endurance athletes (10 skyrunners, 8 road cyclists, and 12 cross-county skiers), Flatt & Esco [24] involved 10 male and 10 female cross-country athletes, and Pereira et al [25] included 35 futsal players. Inclusion of athletes from different sports disciplines may have led to differences in results between the studies. It is also important to note that both studies [24,25] did not specify a formula for the calculation of ICC. There are at least six formulas for calculating an ICC, all yielding different values [31] and this may also explain the differences between the studies.

Previously, it was shown that Ln RMSSD assessment alone may be misleading due to possible presence of the saturation phenomenon, specifically in athletes with very high cardiac vagal activity [22]. In this context, Flatt & Esco [24] unfortunately, did not analyze the stability of either HR or another suitable HRV index. Pereira et al. [25] analyzed the stability of the Ln RMSSD/RR index that could be also used for detecting the saturation phenomenon [22]. However, the evaluation of the Ln RMSSD/RR change is not straightforward, as it can be caused by both the change of numerator (Ln RMSSD) and denominator (RR). Therefore, the current study performed a separate assessment of Ln RMSSD and HR.

The results of this study showed that HR stabilizes earlier than Ln RMSSD and that a 30 s period was sufficient for HR stabilization in endurance trained athletes. The 1 min period required to stabilize the Ln RMSSD was also suitable for HR stabilization. However, the 30 s required to stabilize the HR was not sufficient to stabilize the Ln RMSSD. For example, the ithlete mobile HRV application uses an algorithm to start recording Ln RMSSD based on detecting a stable HR [5]. It is questionable whether this is an accurate approach, because the present study showed that HR stabilizes earlier than Ln RMSSD.

This study also found that the time-effective Ln RMSSD evaluation (1-min stabilization and 1-min recording periods), whilst suitable for athletes, was not valid for university students, because at least 1.5 min was required to stabilize Ln RMSSD in students. It is well known, that endurance athletes exhibit higher autonomic nervous system (ANS) activity presented by greater resting cardiac vagal activity together with a lower resting HR compared with the normal population [1,37]. Therefore, one would suggest that a higher cardiac vagal control in endurance athletes enables faster HR and HRV adjustment to stimuli such as the clinostatic maneuver compared with subjects who exhibit low cardiac vagal activity. This finding raises the question as to how generic the time-effective procedure is. This issue is important for subjects with poor HRV activity, e. g. the elderly, because it is feasible that the stabilization period may be longer than 1.5 min. The use of HRV analysis for monitoring responses to physical activity in the elderly is becoming more important [38]. Practitioners who plan to use HRV analysis for other population groups, with specific ANS activity levels should establish and report their own validation studies to determine the necessary stabilization period.

We prefer using Ln RMSSD rather than RMSSD because the logarithmic transformation enables to correct the skewed probability distribution of RMSSD shifting the probability distribution of Ln RMSSD closer to normal [9,18]. Parametric statistical methods that assume normal distribution can then be used. However, non-transformed RMSSD values have been used in the literature [12,13]. Therefore, we also analyzed the stabilization period for RMSSD. In terms of the shortest stabilization periods, no differences were found between Ln RMSSD and RMSSD in this study.

For statistical analyses we prefer using MBI to NHST based on arguments presented by Buchheit [39]. Nevertheless, the discussion of the advantages and disadvantages of MBI continues. Welsh & Knight [40] showed that MBI is less conservative than NHST and recommended that MBI should not be used. We provided S1–S4 Tables with conventional statistical analysis as an alternative for readers who prefer NHST. Conventional statistical analysis revealed the same findings as MBI for the shortest stabilization periods, with one exception. The lower limit of 95% CI for ICC for RMSSD in athletes after 1-min stabilization period was 0.69. This is below our limit of 0.70 and therefore 1-min stabilization period could not be considered long enough by conventional statistical analysis. In our opinion, 0.69 is still as good as 0.70, and therefore accept that MBI and conventional statistical analyses revealed almost the same results in terms of the shortest stabilization periods.

## Conclusions

When ultra-short-term (60 s) recordings are used to calculate Ln RMSSD and HR in the supine position, the minimal stabilization period required to stabilize both indexes was 60 s for endurance athletes but 90 s for university students.

## Supporting information

S1 Table Conventional statistical analysis for Table 1. (DOCX)

S2 Table Conventional statistical analysis for Table 2. (DOCX)

S3 Table Conventional statistical analysis for Table 3. (DOCX)

S4 Table Conventional statistical analysis for Table 4. (DOCX)

S5 Table Raw data for Table 1. (XLSX)

S6 Table Raw data for Table 2. (XLSX)

S7 Table Raw data for Table 3. (XLSX)

S8 Table Raw data for Table 4. (XLSX)
