# Supplementary material for: Stabilization period before capturing an ultra-short vagal index can be shortened to 60 s in endurance athletes and to 90 s in university students
Source: PLoS One. 2018 Oct 8;13(10):e0205115. doi: 10.1371/journal.pone.0205115 (PMC6175275; doi:10.1371/journal.pone.0205115)
Supplement: S1 Table — (DOCX) [file pone.0205115.s001.docx]

**S1 Table. Statistics of the studied groups.**

| **Variable** | **Athletes** | **Students** |  |  |
| --- | --- | --- | --- | --- |
|  | **Mean ± SD** | **Mean ± SD** | **P** | **ES** |
| Age (years) | 24.1 ± 2.3 | 23.3 ± 1.8 | 0.158 | 0.37 |
| Body mass (kg) | 74.5 ± 6.6 | 79.1 ± 5.3 | 0.004 | -0.78 |
| Body height (cm) | 179.9 ± 3.8 | 183.7 ± 4.1 | 0.001 | -0.97 |
| VO_2_max (ml∙kg^-1^∙min^-1^) | 64.1 ± 6.6 | 52.8 ± 5.1 | 0.001 | 1.91 |
| Ln RMSSD (ms) | 4.43 ± 0.50 | 4.30 ± 0.45 | 0.299 | 0.27 |
| RMSSD (ms) | 95 ± 54 | 81 ± 35 | 0.225 | 0.32 |
| HR (beats∙min^-1^) | 50.8 ± 6.2 | 56.3 ± 6.4 | 0.001 | -0.87 |

SD = standard deviation; P = significance of two-sample t-test; ES = Cohen’s effect size; VO_2_max = maximal oxygen consumption; Ln RMSSD = natural logarithm of root mean square of successive differences between adjacent RR intervals; RMSSD = root mean square of successive differences between adjacent RR intervals; HR = heart rate.
