# Supplementary material for: Stabilization period before capturing an ultra-short vagal index can be shortened to 60 s in endurance athletes and to 90 s in university students
Source: PLoS One. 2018 Oct 8;13(10):e0205115. doi: 10.1371/journal.pone.0205115 (PMC6175275; doi:10.1371/journal.pone.0205115)
Supplement: S2 Table — (DOCX) [file pone.0205115.s002.docx]

**S2 Table. Comparison of the Ln RMSSD values that were calculated from a 1-min segment after various stabilization periods (SP) with reference Ln RMSSD values that were calculated from 5-min segments after a 5-min stabilization period.**

| **SP** | **Mean ± SD** | **Bias;**  **±95% CL** | **P** | **ES** | **TE** | **ICC (95% CI)** |
| --- | --- | --- | --- | --- | --- | --- |
| **(min)** | **(ms)** | **(ms)** |  |  | **(ms)** |  |
| Athletes (n = 30) | | | | | | |
| 0.0 | 4.58 ± 0.52 | 0.15; ±0.11 | 0.011 | 0.30 | 0.21 | 0.79 (0.57 to 0.90) |
| 0.5 | 4.55 ± 0.50 | 0.12; ±0.12 | 0.041 | 0.25 | 0.22 | 0.78 (0.58 to 0.89) |
| 1.0 | 4.45 ± 0.59 | 0.02; ±0.12 | 0.693 | 0.05 | 0.22 | 0.84 (0.69 to 0.92) |
| 1.5 | 4.42 ± 0.60 | -0.01; ±0.09 | 0.883 | -0.01 | 0.17 | 0.90 (0.81 to 0.95) |
| 2.0 | 4.42 ± 0.62 | -0.01; ±0.11 | 0.859 | -0.02 | 0.21 | 0.86 (0.73 to 0.93) |
| 2.5 | 4.46 ± 0.62 | 0.03; ±0.10 | 0.534 | 0.06 | 0.19 | 0.89 (0.78 to 0.94) |
| 3.0 | 4.43 ± 0.58 | 0.00; ±0.08 | 0.992 | 0.00 | 0.16 | 0.92 (0.84 to 0.96) |
| 3.5 | 4.37 ± 0.57 | -0.06; ±0.10 | 0.264 | -0.11 | 0.19 | 0.88 (0.76 to 0.94) |
| 4.0 | 4.36 ± 0.60 | -0.07; ±0.09 | 0.114 | -0.14 | 0.17 | 0.90 (0.81 to 0.95) |
| Ref | 4.43 ± 0.50 |  |  |  |  |  |
| Students (n = 30) | | | | | | |
| 0.0 | 4.59 ± 0.46 | 0.29; ±0.12 | <0.001 | 0.64 | 0.23 | 0.62 (0.10 to 0.84) |
| 0.5 | 4.54 ± 0.50 | 0.24; ±0.11 | <0.001 | 0.53 | 0.22 | 0.71 (0.26 to 0.88) |
| 1.0 | 4.45 ± 0.51 | 0.15; ±0.10 | 0.004 | 0.33 | 0.18 | 0.82 (0.58 to 0.92) |
| 1.5 | 4.36 ± 0.47 | 0.06; ±0.08 | 0.117 | 0.14 | 0.15 | 0.88 (0.77 to 0.94) |
| 2.0 | 4.33 ± 0.48 | 0.03; ±0.08 | 0.507 | 0.06 | 0.16 | 0.89 (0.78 to 0.94) |
| 2.5 | 4.33 ± 0.52 | 0.03; ±0.09 | 0.531 | 0.06 | 0.18 | 0.87 (0.75 to 0.94) |
| 3.0 | 4.30 ± 0.53 | -0.00; ±0.08 | 0.993 | 0.00 | 0.16 | 0.90 (0.80 to 0.95) |
| 3.5 | 4.29 ± 0.53 | -0.01; ±0.08 | 0.786 | -0.02 | 0.15 | 0.91 (0.81 to 0.95) |
| 4.0 | 4.26 ± 0.52 | -0.04; ±0.08 | 0.353 | -0.08 | 0.15 | 0.90 (0.80 to 0.95) |
| Ref | 4.30 ± 0.45 |  |  |  |  |  |

SD = standard deviation; Bias = mean difference between the 1-min segment value and reference value; CL = confidence limit; P = significance of one-sample t-test; ES = Cohen’s effect size; TE = typical error; ICC = intraclass correlation coefficient; CI = confidence interval.
