# Supplementary material for: Stabilization period before capturing an ultra-short vagal index can be shortened to 60 s in endurance athletes and to 90 s in university students
Source: PLoS One. 2018 Oct 8;13(10):e0205115. doi: 10.1371/journal.pone.0205115 (PMC6175275; doi:10.1371/journal.pone.0205115)
Supplement: S3 Table — (DOCX) [file pone.0205115.s003.docx]

**S3 Table. Comparison of the RMSSD values that were calculated from a 1-min segment after various stabilization periods (SP) with reference RMSSD values that were calculated from 5-min segments after a 5-min stabilization period.**

| **SP** | **Mean ± SD** | **Bias;**  **±95% CL** | **P** | **ES** | **TE** | **ICC (95% CI)** |
| --- | --- | --- | --- | --- | --- | --- |
| **(min)** | **(ms)** | **(ms)** |  |  | **(ms)** |  |
| Athletes (n = 30) | | | | | | |
| 0.0 | 112 ± 63 | 16; ±12 | 0.012 | 0.30 | 23 | 0.81 (0.60 to 0.91) |
| 0.5 | 107 ± 58 | 12; ±12 | 0.060 | 0.22 | 24 | 0.81 (0.63 to 0.91) |
| 1.0 | 102 ± 66 | 7; ±11 | 0.220 | 0.12 | 20 | 0.89 (0.78 to 0.94) |
| 1.5 | 100 ± 67 | 4; ±8 | 0.284 | 0.08 | 15 | 0.94 (0.87 to 0.97) |
| 2.0 | 101 ± 70 | 5; ±11 | 0.303 | 0.10 | 20 | 0.90 (0.80 to 0.95) |
| 2.5 | 105 ± 71 | 9; ±10 | 0.078 | 0.17 | 20 | 0.89 (0.79 to 0.95) |
| 3.0 | 99 ± 61 | 3; ±8 | 0.380 | 0.06 | 15 | 0.93 (0.87 to 0.97) |
| 3.5 | 93 ± 58 | -2; ±10 | 0.614 | -0.05 | 19 | 0.89 (0.79 to 0.95) |
| 4.0 | 93 ± 61 | -2; ±7 | 0.540 | -0.04 | 13 | 0.95 (0.90 to 0.98) |
| Ref | 95 ± 54 |  |  |  |  |  |
| Students (n = 30) | | | | | | |
| 0.0 | 108 ± 45 | 27; ±13 | <0.001 | 0.78 | 24 | 0.52 (0.07 to 0.77) |
| 0.5 | 105 ± 47 | 24; ±11 | <0.001 | 0.68 | 21 | 0.64 (0.18 to 0.84) |
| 1.0 | 96 ± 48 | 15; ±10 | 0.004 | 0.44 | 19 | 0.74 (0.45 to 0.88) |
| 1.5 | 87 ± 37 | 6; ±7 | 0.095 | 0.16 | 13 | 0.87 (0.74 to 0.94) |
| 2.0 | 84 ± 38 | 3; ±6 | 0.314 | 0.09 | 12 | 0.89 (0.78 to 0.94) |
| 2.5 | 86 ± 43 | 5; ±8 | 0.227 | 0.14 | 16 | 0.84 (0.69 to 0.92) |
| 3.0 | 84 ± 42 | 3; ±8 | 0.494 | 0.07 | 14 | 0.86 (0.73 to 0.93) |
| 3.5 | 83 ± 41 | 2; ±7 | 0.594 | 0.05 | 13 | 0.89 (0.78 to 0.95) |
| 4.0 | 81 ± 42 | -0; ±7 | 0.933 | -0.01 | 14 | 0.88 (0.76 to 0.94) |
| Ref | 81 ± 35 |  |  |  |  |  |

SD = standard deviation; Bias = mean difference between the 1-min segment value and reference value; CL = confidence limit; P = significance of one-sample t-test; ES = Cohen’s effect size; TE = typical error; ICC = intraclass correlation coefficient; CI = confidence interval.
