# Supplementary material for: Stabilization period before capturing an ultra-short vagal index can be shortened to 60 s in endurance athletes and to 90 s in university students
Source: PLoS One. 2018 Oct 8;13(10):e0205115. doi: 10.1371/journal.pone.0205115 (PMC6175275; doi:10.1371/journal.pone.0205115)
Supplement: S4 Table — (DOCX) [file pone.0205115.s004.docx]

**S4 Table. Comparison of heart rate (HR) values that were calculated from a 1-min segment after various stabilization periods (SP) with reference HR values that were calculated from 5-min segments after a 5-min stabilization period.**

| **SP** | **Mean ± SD** | **Bias;**  **±95% CL** | **P** | **ES** | **TE** | **ICC (95% CI)** |
| --- | --- | --- | --- | --- | --- | --- |
| **(min)** | **(ms)** | **(ms)** |  |  | **(ms)** |  |
| Athletes (n = 30) | | | | | | |
| 0.0 | 53.3 ± 7.5 | 2.6; ±1.4 | 0.001 | 0.42 | 2.7 | 0.79 (0.47 to 0.91) |
| 0.5 | 50.6 ± 7.1 | -0.2; ±1.2 | 0.721 | -0.03 | 2.2 | 0.89 (0.78 to 0.95) |
| 1.0 | 50.2 ± 6.9 | -0.6; ±0.9 | 0.206 | -0.09 | 1.7 | 0.93 (0.87 to 0.97) |
| 1.5 | 50.0 ± 6.9 | -0.8; ±0.8 | 0.060 | -0.12 | 1.5 | 0.94 (0.88 to 0.97) |
| 2.0 | 50.0 ± 7.0 | -0.8; ±0.8 | 0.056 | -0.13 | 1.6 | 0.93 (0.86 to 0.97) |
| 2.5 | 50.1 ± 6.6 | -0.6; ±0.7 | 0.068 | -0.11 | 1.3 | 0.95 (0.90 to 0.98) |
| 3.0 | 50.3 ± 6.3 | -0.5; ±0.9 | 0.292 | -0.07 | 1.6 | 0.93 (0.86 to 0.97) |
| 3.5 | 50.7 ± 6.7 | -0.1; ±0.9 | 0.912 | -0.01 | 1.8 | 0.93 (0.85 to 0.96) |
| 4.0 | 50.9 ± 7.1 | 0.1; ±1.1 | 0.796 | 0.02 | 2.0 | 0.91 (0.82 to 0.96) |
| Ref | 50.8 ± 6.2 |  |  |  |  |  |
| Students (n = 30) | | | | | | |
| 0.0 | 58.5 ± 6.3 | 2.2; ±2.0 | 0.034 | 0.35 | 3.9 | 0.60 (0.30 to 0.79) |
| 0.5 | 56.5 ± 6.5 | 0.2; ±1.8 | 0.835 | 0.03 | 3.4 | 0.72 (0.49 to 0.86) |
| 1.0 | 55.2 ± 6.1 | -1.1; ±1.1 | 0.063 | -0.17 | 2.1 | 0.87 (0.75 to 0.94) |
| 1.5 | 55.6 ± 6.1 | -0.7; ±0.8 | 0.087 | -0.11 | 1.6 | 0.93 (0.86 to 0.97) |
| 2.0 | 56.0 ± 5.9 | -0.3; ±0.8 | 0.434 | -0.05 | 1.5 | 0.94 (0.88 to 0.97) |
| 2.5 | 56.0 ± 6.1 | -0.2; ±0.8 | 0.562 | -0.03 | 1.5 | 0.95 (0.89 to 0.97) |
| 3.0 | 55.8 ± 6.2 | -0.5; ±0.7 | 0.152 | -0.07 | 1.2 | 0.96 (0.92 to 0.98) |
| 3.5 | 55.7 ± 6.0 | -0.5; ±0.6 | 0.074 | -0.09 | 1.1 | 0.96 (0.92 to 0.98) |
| 4.0 | 55.8 ± 6.2 | -0.5; ±0.7 | 0.154 | -0.08 | 1.3 | 0.96 (0.91 to 0.98) |
| Ref | 56.3 ± 6.4 |  |  |  |  |  |

SD = standard deviation; Bias = mean difference between the 1-min segment value and reference value; CL = confidence limit; P = significance of one-sample t-test; ES = Cohen’s effect size; TE = typical error; ICC = intraclass correlation coefficient; CI = confidence interval.
